# Book Reviews

**Published:** 1909-03

**Authors:** 


### BOOK REVIEWS

WOMAN. A Treatise on the Normal and Pathological Emotions of Feminine Love. By Bernard S. Talmy, M. D., Gynecologist to the Yorkville Hospital and Dispensary, former Pathologist to the Mothers' and Babies' Hospital, etc., New York. For Physicians and Students of Medicine, with 23 drawings. Fourth enlarged and revised edition. Published by the Practitioners' Publishing Co., New York.

Rarely now-a-days do books fill what may be truly said to be a "long felt want;" occasionally such is the case and "Woman" has proved that it falls in the class of such works by the call for new editions every few months.

Talmy has made a careful study of this intricate subject and has presented to the public a clear, concise account of this little-discussed yet important subject. Many homes have doubtless been wrecked by the ignorance of physicians, who when consulted regarding these matters, could not deal with them in a scientific manner.

The book is divided into 8 parts which are devoted to the following subjects: I Introduction, II Evolution of Sex, II Anatomy of the Genitals, IV Physiology, V Pathology, VI Hygiene, VII Psychology, VIII Morality.

The spirit in which this book is written removes from it the smutty or vulgar interpretations that might be placed upon a subject of such delicate nature. A clear understanding of woman is necessary for all physicians and we commend to them this book.

---

BACTERIAL FOOD POISONING. A concise exposition of the etiology, bacteriology, pathology, symptomotology, prophylaxis, and treatment of so-called ptomaine poisoning. By Prof. Dr. A. Dieudonne, Munich. Translated and edited, with additions, by Dr. Charles F. Boldouan, Bacteriologist, Research Laboratory, Department of Health, City of New York. Authorized translation. Published by E. B. Treat & Co., New York. Price $1.00.

This practical little book should prove of great value to health officers who are so often called upon to investigate poisoning by food. The favorable reception of the original work by Dieudonne made the succes sof a translation almost certain; Boldouan has well performed his task as editor and has incorporated additional outbreaks of food poisoning and has adopted the work to American conditions.

Much greater importance is now placed upon the bacterial nature of these poisons than formerly when they were thought to be due to "ptomaines." We can cheerfully recommend the work to all who are interested in such subjects.

---

ORTHOPEDIC SURGERY FOR PRACTITIONERS. By Henry Long Taylor, M. D., Prof. of Orthopedic Surgery and Attending Orthopedic Surgeon, New York Post-Graduate Medical School and Hospital; Assistant Surgeon, Hospital for the Ruptured and Crippled, New York. Assisted by Charles Ogilvy, M. D., Adjunct Prof. of Orthopedic Surgery, New York Post-Graduate Medical School and Hospital etc., and by Fred H. Albee, M. D., Instructor in Orthopedic Surgery, New York Post-Graduate Medical School and Hospital, New York. With 254 illustrations. D. Appleton & Co., New York, Publishers.

Taylor has succeeded remarkably well in carrying out his plan of presenting this subject in a form suitable for the general practitioner, who sees, oftener than the specialist, the crippling affections in their incipiency when perhaps comparatively simple methods of treatment may save many from deformity or death.

The work is divided into general, special and technical parts; this arrangement economizes space, emphasizes the importance of underlying causes and is more convenient for reference. In the general part in which the underlying principles are discussed the reviewer looked for reference to the excellent work on intestinal proteid putrifaction which has been done by Hoke and Andrews, but failed to find any mention of the possibility of such a cause for certain forms of rheumatism. That this is a fundamental cause of many joint affections has been clearly demonstrated by curing the patients by proper regulation of diet and bowels, with results, at times, quite remarkable.

Taylor has had twenty-five years' experience in orthopedic work and in his book gives the gist of it to the practitioner in an interesting and attractive form. The book is beautifully printed and well bound, and the illustrations are good, most of them having been taken especially for this work.

---

TRANSACTIONS OF TENTH ANNUAL MEETING OF THE AMERICAN PRACTOLOGIC SOCIETY. Edited by Samuel T. Earle, M. D., and Lewis H. Adler, Jr., M. D.

This volume contains the papers and discussions read at the 1908 meeting of the society, together with a list of its members and a note as to the date of its organization, as well as a list of the meeting places and of its officers from the inception of its organization to the present time.

Many excellent contributions to rectal surgery are contained in this book, by many of the foremost men in America.

---

CLINICAL DIAGNOSIS AND TREATMENT OF DISORDERS OF THE BLADDER. With Technique of Cystoscopy. By Follen Cabot, M. D., Prof. of Genito-Urinary Diseases, Post-Graduate Medical School; Attending Genito-Urinary Surgeon, City and Post-Graduate Hospitals, New York, Illustrated. Published by E. B. Treat & Co., New York. 8 vo. 225 pages, prepaid $2.00.

This well written little volume on diseases of the bladder by Cabot affords the practitioner a clear and safe guide in the management of these disorders. The extensive clinical experience and the careful work done by the author well qualify him to write the book we have under review. Attention is given to case recording, management of the patient and the methods of examination necessary to elicit the facts required for the diagnosis.

Especial attention is appropriately given to cystoscopy, but other useful methods of diagnosis have also been discussed. The book is fairly well illustrated and is well printed and bound.

E. G. B.

---

APPENDICITIS and Other Diseases of the Vermiform Appendix. By Howard A. Kelly, M. D., with 215 original illustrations, some in colors and three lithographic plates. Published by J. B. Lippincott Co., Philadelphia. $6.00 net.

So excellent have been the previous contributions to medical literature by Howard Kelly that we have come to look upon his work as among the best that we have the pleasure of reviewing. This monograph on the appendix is fully up to the standard set by Kelly's other books, and will long remain a storehouse from which surgeons may acquire the most reliable information upon this important and widely studied organ. The graphic illustrations by Miss Ruth Huntington, now Mrs. Max Brodel, deserve especial commendation, as also do the printing and binding by these well known publishers.

Kelly lays much stress on promptitude in operating, and urges that time be not lost by dawdling when the necessity for operation is clearly recognized; golden moments may be lost and result in a fatal result when prompt action would have saved the patient's life.

Much attention is devoted to pathology, clinical history, treatment previous to operation, operative treatment, and care of the patient after operation and post-operative selquelae.

The earliest definite anatomical record of disease of the appendix appears to have been made by Lawrence Heister in 1711 and reported in 1755; the first reported cases of appendicitis observed during life is the one of Mestivier in 1759.

The general excellence of this work justifies its recommendation without reservation, as its teachings are safe, thorough and attractively presented.

---

COSMETIC SURGERY. The correction of featural imperfections. By. Charles C. Miller, M. D., Second Edition Enlarged.

Including the description of numerous operations for improving the appearance of the face. 160 pages. 96 illustrations. Prepaid $1.50. Published by the author, 70 State street, Chicago.

